# Co-exposure to zymosan A and heat-inactivated Asian sand dust exacerbates ovalbumin-induced murine lung eosinophilia

**DOI:** 10.1186/s13223-016-0153-x

**Published:** 2016-10-10

**Authors:** Kaori Sadakane, Takamichi Ichinose, Masataka Nishikawa, Hirohisa Takano, Takayuki Shibamoto

**Affiliations:** 1 Department of Health Sciences, Oita University of Nursing and Health Sciences, Oita, 870-1201 Japan; 2 Environmental Chemistry Division, National Institute for Environmental Studies, Tsukuba, Ibaraki 305-8506 Japan; 3 Environmental Health Division, Department of Environmental Engineering, Graduate School of Engineering, Kyoto University, Kyoto, 615-8530 Japan; 4 Department of Environmental Toxicology, University of California, Davis, CA 95616 USA

**Keywords:** Zymosan A, Asian sand dust, Ovalbumin, Eosinophils, Th2, IgE

## Abstract

**Background:**

Epidemiological studies have implicated Asian sand dust (ASD) in the increased prevalence of respiratory disorders, including asthma. It has been observed that fungal elements such as β-glucan can be adsorbed onto ASD. In the present study, the exacerbating effect of the combined exposure to zymosan A (ZymA) containing yeast β-glucan and heat-inactivated ASD on ovalbumin (OVA)-induced murine lung eosinophilia was investigated.

**Methods:**

BALB/c mice were repeatedly instilled intratracheally with one of eight immunogenic formulations consisting of various combinations of (1) ZymA, (2) ASD that was briefly heated to remove organic substances (H-ASD), and (3) OVA in normal saline, or each of the above alone. Pathologic changes, cytological alterations in bronchoalveolar lavage fluid (BALF), changes in inflammatory cytokines and chemokines in BALF, and OVA-specific IgE and IgG_1_ antibodies in serum were investigated.

**Results:**

Exposure to ZymA with or without OVA had no effect on most indicators of lung inflammation. Exposure to H-ASD with OVA increased the recruitment of inflammatory cells to the lungs and the serum levels of OVA-specific IgE and IgG_1_. The combination OVA + ZymA + H-ASD induced a marked recruitment of eosinophils and upregulation of T helper 2 (Th2) cytokines (interleukin [IL]-4 and IL-13), IL-6, eotaxin/CCL11, and monocyte chemotactic protein (MCP)-3/CCL7 in BALF and OVA-specific IgE in serum. This treatment also induced the most severe pathological changes in the lungs of mice. ZymA was found to boost the effects of H-ASD, thereby exacerbating the OVA-induced allergic inflammation, even though ZymA alone did not have such effect.

**Conclusions:**

The results suggest that fungal elements such as β-1,3-glucan aggravate the allergic inflammation caused by ASD. Our findings may facilitate prophylaxis of some allergic diseases in Asia.

## Background

Asian sand dust (ASD) is a major source of air pollution in East Asia, including East China, the Korean Peninsula, Taiwan, and Japan, in spring and early summer [1–4]. Recently, other deleterious effects, including damage to food crops and human health, have been uncovered. In particular, the effects of ASD on human health have become more serious. Epidemiological studies have implicated ASD in the increased prevalence of respiratory disorders, including asthma [5–7]. Furthermore, the increase in the rate of deaths caused by respiratory and cardiovascular diseases may be associated with ASD pollution [8–10]. Our previous studies have shown that ASD exacerbates ovalbumin (OVA)-induced murine lung eosinophilia [11, 12], suggesting that ASD is an exacerbation factor of allergic respiratory diseases. However, it was unclear which component of the ASD is responsible for this effect because the wind-borne ASD is not simply composed of sand dust particles but includes various other components such as microorganisms and chemicals [13, 14].

One study showed that two types of ASD sampled during different periods, in different source regions, and via different passage routes, caused different pathological changes in a mouse model of allergic asthma [15]. Analysis of the composition of the two ASD types revealed that the ASD rich in microorganisms aggravated the allergic inflammation more strongly. Moreover, ASD heated at 360 °C for 30 min to eliminate organic substances (H-ASD) has fewer adverse effects [12]. Taken together, these findings suggested that microorganisms in ASD may exacerbate allergic airway inflammation.

Eight strains of microorganisms have been previously identified in an ASD aerosol collected at an altitude of 400 m over the Noto peninsula facing the Sea of Japan [13]. One of the microorganisms identified, the fungus *Bjerkandera adusta*, together with H-ASD, strongly exacerbated OVA-induced murine lung eosinophilia [16]. *Bjerkandera adusta* is a fungus that mostly colonizes rotting wood [17]. It produces abundant asexual spores from the hyphae [18]. The size of the spores is 4–5 µm [19], which is the approximately the same size as that of ASD. In the above studies, fragments of hyphae and spores sonicated with an ultrasonic disrupter were used. However, other studies have shown that the fungal elements are actually adsorbed onto ASD [20].

In a recent paper, we reported that ASD induces Toll-like receptor (TLR)2 and TLR4 signals to trigger T helper 2 (Th2)-dominant lung allergic inflammation via a myeloid differentiation factor 88 (MyD88)-dependent signaling pathway [21]. TLRs are the principal innate immune sensors recognizing microbial pathogen-associated molecular patterns from bacterial, fungal, and viral structures [22]. The TLR4 ligand lipopolysaccharide (LPS) and TLR2 ligands such as β-glucan are strong candidates for causing the exacerbation of lung eosinophilia by ASD [21]. An in vitro study showed that TLR2 rather than TLR4 contributes to the production of pro-inflammatory cytokines from bone marrow-derived macrophages [23]. On the basis of these results, we speculated that ASD-adherent β-glucan is one of the exacerbating factors of lung eosinophilia.

In the present study, the exacerbating effects of the combined treatment with commercial zymosan A (ZymA) from the yeast *Saccharomyces cerevisiae*, as a source of β-glucan, and H-ASD on OVA-induced lung eosinophilia were investigated in a mouse model of asthma. The ASD was heated at 360 °C to exclude toxic materials such as microbial materials including β-glucan, prior to application. Our investigation included the examination of pathologic changes, cytological alterations in bronchoalveolar lavage fluid (BALF), changes in inflammatory cytokines and chemokines in BALF, and OVA-specific immunoglobulin (Ig)E and IgG_1_ antibodies in the serum.

## Methods

### Animals

One hundred and twelve male BALB/c mice (7 weeks old), born and reared under specific pathogen-free conditions and weighing between 21 and 26 g, were purchased from Charles River Japan (Kanagawa, Japan). The mice were acclimated for 1 week at a facility maintained at 23–25 °C and 50–70 % relative humidity under conventional conditions (6–8 mice per cage, a 12/12-h light/dark cycle, and ad libitum access to water and a commercial diet [CE-2; Japan Clea Co., Tokyo, Japan]). The animals were treated humanely and every effort was made to reduce their suffering, in accordance with the animal care methods that had been approved by the Animal Care and Use Committee of the Oita University of Nursing and Health Sciences, Oita, Japan.

### Preparation of the particles

National Institute for Environmental Study (NIES) No. 30 “Gobi Kosa Dust” was used in the present study for preparation of the ASD samples. This ASD was collected from surface soils in the Gobi Desert in Mongolia. The aerodynamic diameter (median diameter) of the dust was approximately 4 μm, which is within the range of the median diameter (3–5 μm) of the Asian mineral dust that is wind-borne to Japan. The chemical composition (elements) of the particles has been reported previously [24]. The certified data on NIES No. 30 are as follows: 0.939 % ± 0.071 % Na, 1.51 % ± 0.13 % Mg, 7.58 % ± 0.42 % Al, 2.13 % ± 0.11 % K, 4.25 % ± 0.35 % Ca, 0.426 % ± 0.040 % Ti, 3.84 % ± 0.35 % Fe, 768 ± 83 mg/kg Mn, 93.1 ± 8.5 mg/kg Zn, 250 ± 20 mg/kg Sr, 535 ± 31 mg/kg Ba; the reference data for this dust are 24.1 % Si, 955 mg/kg P, 13.1 mg/kg Sc, 57.4 mg/kg Cr, 13.7 mg/kg Co, 29.1 mg/kg Ni, 34.1 mg/kg Cu, 40.4 mg/kg La, 22.4 mg/kg Pb, 13.0 mg/kg Th, and 2.62 mg/kg U [14]. The ASD was heated at 360 °C for 30 min in an electric heater to eliminate organic matter and toxic materials (e.g., LPS, β-glucan, sulfate, nitrate, microorganisms). The resultant heat-inactivated ASD is called *H*-*ASD* in this study.

### Reagents and analysis of ZymA

We used Zymosan A (cat. #Z4250) from *S. cerevisiae* purchased from Sigma-Aldrich Co. (St. Louis, MO, USA) as a ligand for TLR2. The content of β-glucan in ZymA was measured using a β-1,3-d-glucan detection reagent kit (Associates of Cape Cod, Inc., MA, USA). Grade VII OVA, the allergen that was used to induce allergic airway inflammation, was also purchased from Sigma-Aldrich Co.

### Study protocol

The mice were divided into eight treatment groups (n = 14 per group) as follows: (1) control, (2) ZymA, (3) H-ASD, (4) ZymA + H-ASD, (5) OVA, (6) OVA + ZymA, (7) OVA + H-ASD, (8) OVA + ZymA + H-ASD. The dose of ZymA was 20 ng per mouse, the dose of H-ASD was 0.1 mg per mouse, and the dose of OVA was 4 µg per mouse. ZymA, H-ASD, OVA, and the combinations thereof were dissolved or suspended in 0.1 ml each in sterile Otsuka normal saline (Otsuka Pharmaceutical Co., Ltd., Tokyo, Japan). The mice were intratracheally administrated with the mixed or individual solutions four times at 2-week intervals. Animals in the control group received an intratracheal administration of 0.1 ml of sterile saline.

### Analysis of BALF

Eight out of the 14 mice in each group were examined for free-cell content in BALF. These fluid and cell counts were analyzed using a previously reported method [25]. Briefly, the lungs were lavaged with two injections of 0.8 ml of sterile saline at 37 °C. After the fluids from the first and second lavage were mixed and cooled on ice, the resultant solution was centrifuged at 210×*g* for 10 min at 4 °C. The BALF supernatant was stored at −80 °C until analysis of cytokines and chemokines.

The total number of inflammatory cells in BALF was determined in the fresh precipitate by means of a hemocytometer. Cell counts were also determined on cytological preparations. The slides were prepared with a Cytospin (Sakura Finetek Japan, Tokyo, Japan) and stained with Diff-Quik (Sysmex Co., Hyogo, Japan) to identify the eosinophils as red granules. A total of 300 cells in each sample were analyzed under a microscope.

### Histopathological examination

The remaining six mice in each group were used for histopathological examination. Their lungs were fixed with zinc fixative (BD Biosciences, Franklin Lakes, NJ, USA). After separation of the lobes, 2-mm-thick blocks were taken for paraffin embedding. The embedded blocks were sectioned (thickness 3 μm), and the slices were stained with alcian blue (AB) to evaluate the degree of mucus secretion in the bronchial epithelium (from proximal to distal parts). To determine the number of eosinophils in the submucosa of the airway, the slices were also stained with a slightly modified original luna stain [26]. The slices were immersed in a hematoxylin solution and then in Biebrich scarlet solution for 15 and 20 min, respectively. After a rinse with tap water, the slices were dipped in 1 % hydrochloric acid (in ethanol) six times, followed by five dips in a 0.5 % lithium carbonate solution. Five randomly selected visual fields were photographed by means of a light microscope (400× magnification). The number of eosinophils was determined. Histopathological examination of inflammatory cells and epithelial cells in the airway was performed under a Nikon ECLIPSE light microscope (Nikon Co., Tokyo, Japan).

### Quantitation of cytokines and chemokines in BALF

Protein levels of cytokines and chemokines were determined by enzyme-linked immunosorbent assays (ELISAs). Interleukin (IL)-1β, IL-4, IL-6, IL-13, interferon (IFN)-γ, eotaxin/CCL11, keratinocyte chemoattractant (KC)/CXCL1, monocyte chemotactic protein (MCP)-1/CCL2, and macrophage inflammatory protein (MIP)-1α/CCL3 were quantified using ELISA kits from R&D Systems Inc. (Minneapolis, MN, USA). IL-5 and IL-12 were quantified using an ELISA kit from Endogen, Inc. (Woburn, MA, USA). MCP-3/CCL7 levels were measured using an ELISA kit from Bender MedSystems Inc. (Burlingame, CA, USA). The detection limits of IL-1β, IL-4, IL-6, IL-13, IFN-γ, eotaxin/CCL11, KC/CXCL1, MCP-1/CCL2, MIP-1α/CCL3, IL-5, IL-12, and MCP-3/CCL7 were 2.31, 2, 1.6, 1.5, 2, 3, 2.0, 2, 1.5, 5, 12, and 2.6 pg/ml, respectively.

### OVA-specific IgE and IgG_1_ antibodies in serum

These antibodies were quantified using the Mouse OVA-IgE ELISA Kit and Mouse OVA-IgG_1_ ELISA Kit (Shibayagi Co., Gunma, Japan). According to the manufacturer’s protocol, 1 U/ml of anti-OVA IgE was defined as 1.3 ng/ml of the antibody, and 1 U/ml of anti-OVA IgG_1_ was defined as 160 ng/ml of the antibody. Absorbance at 450 nm (sub-wavelength, 620 nm) for OVA-specific IgE and IgG_1_ antibodies was measured on a microplate reader (Bio-Rad Laboratories, Hercules, CA, USA).

### Statistical analysis

All calculations were performed in the SPSS Statistics ver. 23 (IBM, Armonk, NY, USA), and the results are reported as the mean ± standard error. All data were tested by one-way analysis of variance (ANOVA) followed by Tukey’s honestly significant difference test. A *p* value < 0.05 was considered significant.

## Results

### Concentration of β-glucan in ZymA

The detectable content of β-glucan in ZymA was 134 pg/ng (13.4 %).

### Co-exposure to ZymA and H-ASD increased eosinophil infiltration into the lung according to analysis of BALF

**Fig. 1:**
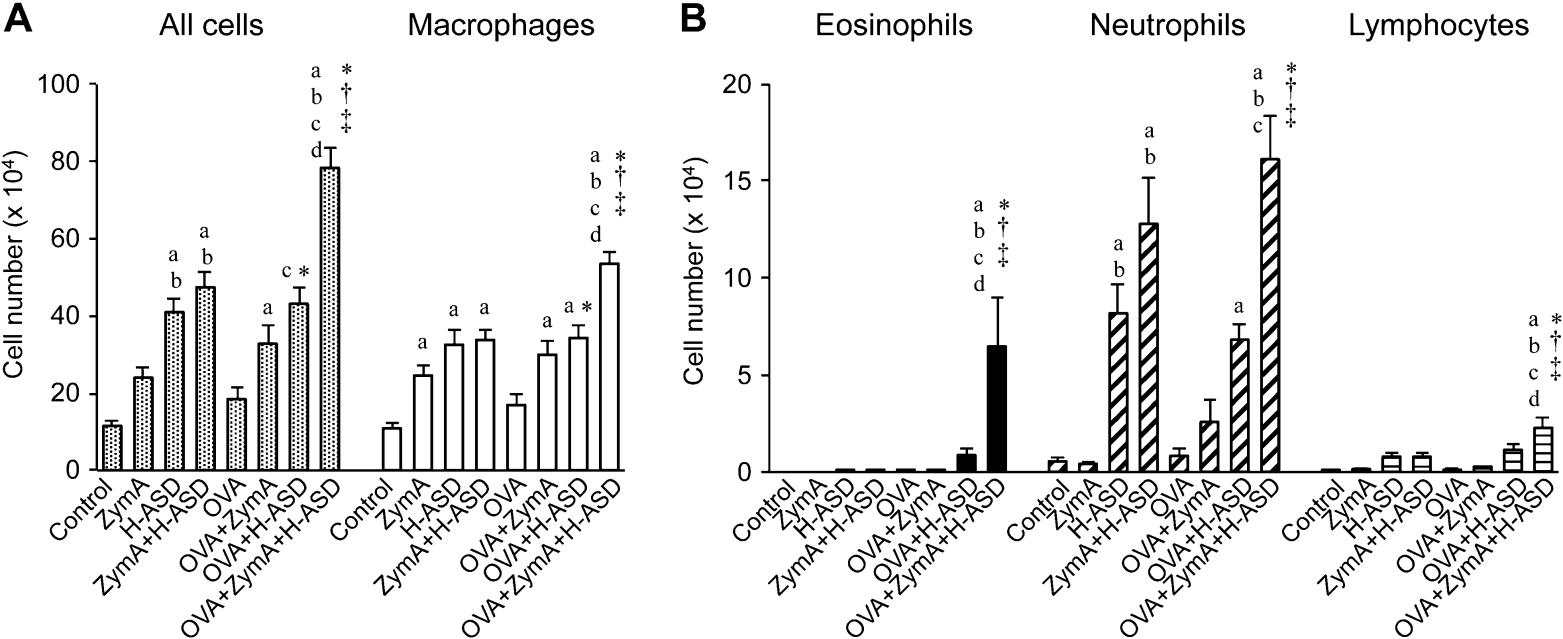
Profiles of inflammatory cells in BALF. All data are expressed as the mean ± SE, n = 8 per group.** A** Total number of inflammatory cells and the number of macrophages in BALF.** B** Number of eosinophils, neutrophils, and lymphocytes in BALF.* a* *p* < 0.05 vs. control,* b* p < 0.05 vs. ZymA,* c* p < 0.05 vs. H-ASD,* d* p < 0.05 vs. ZymA + H-ASD, *p < 0.01 vs. OVA, ^†^p < 0.05 vs. OVA + ZymA, ^‡^p < 0.05 vs. OVA + H-ASD

Figure 1 shows the cellular profiles of the BALF samples. Groups ZymA and OVA did not show a significant increase in total cell number as compared to the control group, whereas the other groups showed a marked increase in total cells (Fig. 1A). H-ASD enhanced the infiltration by inflammatory cells according to the BALF analysis. In contrast, the addition of ZymA to H-ASD did not increase the total cell number over that of the H-ASD-only group. The addition of ZymA to OVA increased the total cell number as compared to OVA alone but the difference was not statistically significant. The addition of H-ASD to OVA significantly increased the total cell number in BALF in comparison with OVA alone. The combination of ZymA, H-ASD, and OVA significantly increased the number of inflammatory cells in BALF as compared to the OVA, OVA + ZymA, and OVA + H-ASD groups. Changes in macrophages showed a trend similar to those in total cells in all groups except the OVA + ZymA group, which showed a significant increase over the OVA-only group.

The number of eosinophils was not increased by the administration of ZymA alone, H-ASD alone, or OVA alone (Fig. 1B). Moreover, the combination ZymA + OVA did not increase the number of eosinophils, and neither did the combination H-ASD + OVA. Only ZymA + OVA + H-ASD caused a marked increase in comparison to the other groups (Fig. 1B); this was also the case for the number of lymphocytes in BALF. The administration of H-ASD increased the number of neutrophils; moreover, the addition of ZymA to H-ASD exacerbated this effect with or without OVA.

### Co-exposure to ZymA and H-ASD exacerbated OVA-induced pathological alterations in the lungs

**Fig. 2:**
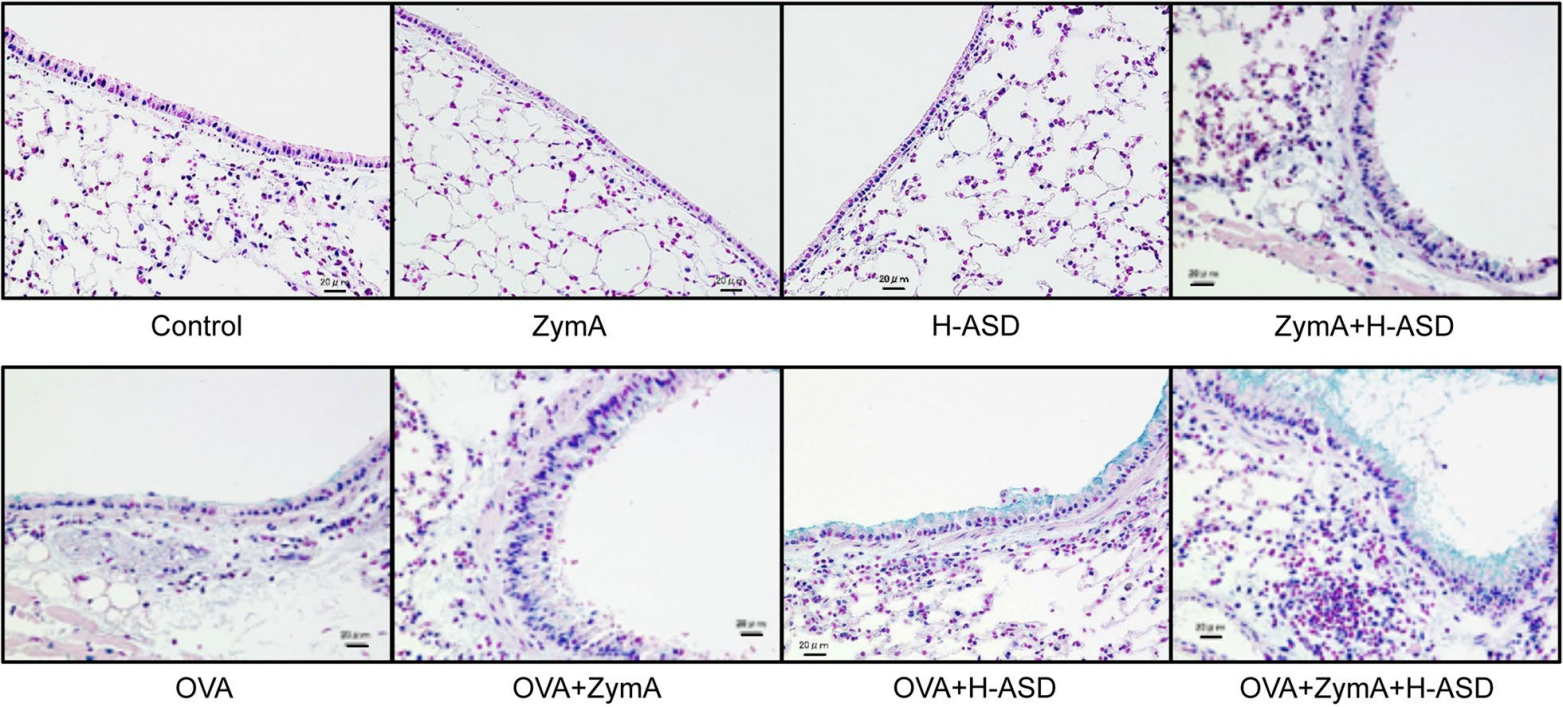
Effects of ZymA and H-ASD on pathological changes in lungs stained with Alcian blue. The* scale bar* is 20 µm

AB staining of the mouse lungs is presented in Fig. 2. No pathological changes were detected in the lungs of the control, ZymA, or H-ASD groups, whereas the combination ZymA + H-ASD caused mild to moderate infiltration of the airway by inflammatory cells and slight proliferation of goblet cells in the airway epithelium. OVA alone caused slight infiltration of the airway submucosa by inflammatory cells as well as mucus production by the airway epithelium. The pathological changes in the OVA + ZymA group were somewhat stronger than those in the OVA group. The combination OVA + H-ASD caused moderate inflammatory cell infiltration of the airway submucosa by as well as moderate proliferation of goblet cells. In contrast, the combination OVA + ZymA + H-ASD induced the greatest production of excess mucus and proliferation of goblet cells in the airway epithelium, and marked accumulation of inflammatory cells in the airway submucosa.

**Fig. 3:**
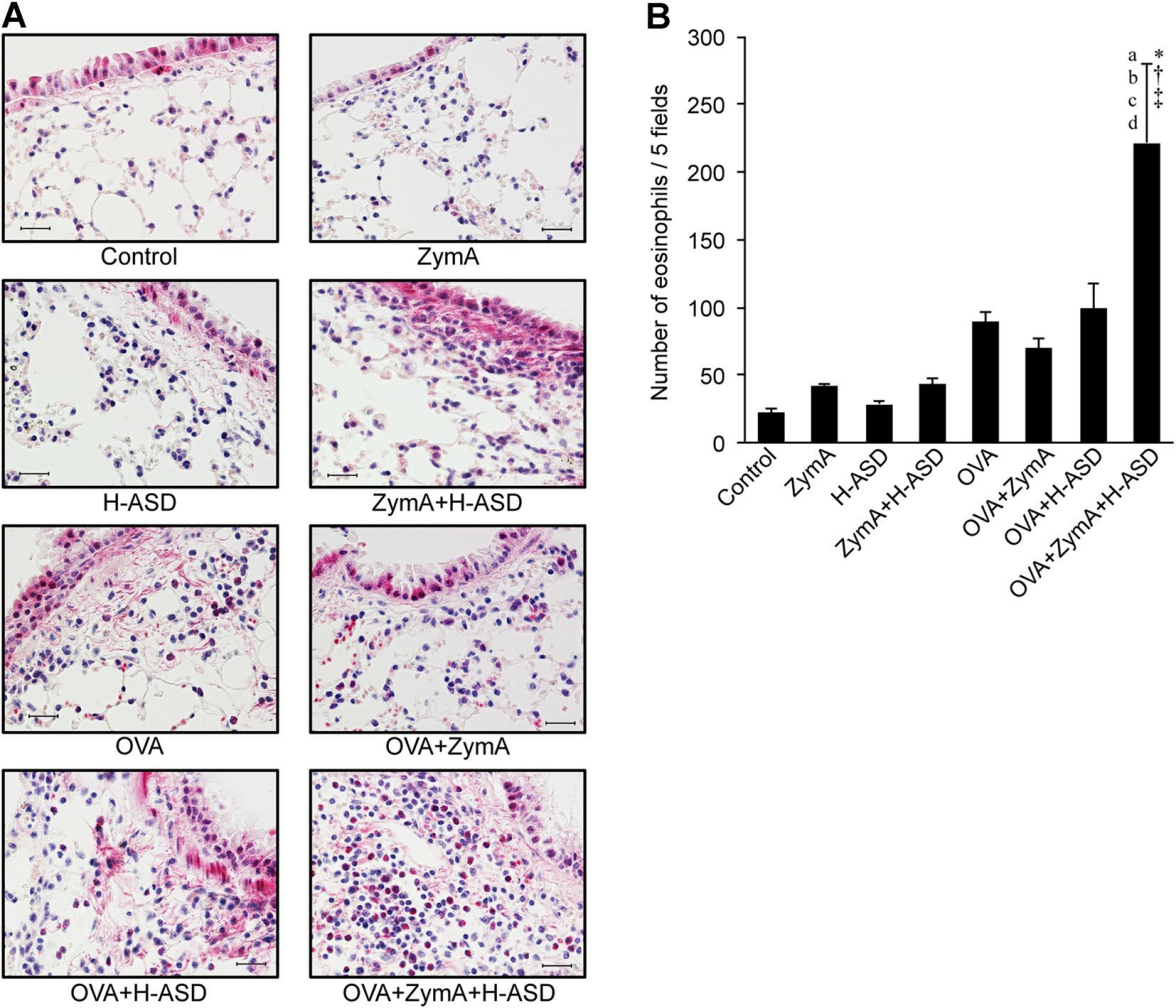
Eosinophilic infiltration of the lung submucosa.** A** Microscopic images of eosinophils stained red with the Luna dye. The* scale bar* is 20 µm.** B** Number of eosinophils in the lung submucosa. All data are expressed as the mean ± SE, n = 6 per group.* a* p < 0.05 vs. control,* b* p < 0.05 vs. ZymA,* c* p < 0.05 vs. H-ASD,* d* p < 0.05 vs. ZymA + H-ASD, *p < 0.05 vs. OVA, ^†^p < 0.05 vs. OVA + ZymA, ^‡^p < 0.05 vs. OVA + H-ASD

Figure 3 shows the presence of eosinophils in the lung submucosa. The greatest accumulation of eosinophils was observed in the OVA + ZymA + H-ASD group (Fig. 3A). Exposure to ZymA, H-ASD, or ZymA + H-ASD yielded the same number of eosinophils as that in the control group (Fig. 3B). In the OVA, OVA + ZymA, and OVA + H-ASD groups, we observed a moderate increase in eosinophils, ranging from 69.8 ± 6.8 cells/5 fields (OVA + ZymA) to 98.8 ± 18.0 cells/5 fields (OVA + H-ASD). In the OVA + ZymA + H-ASD group, eosinophils were significantly increased (221.7 ± 57.8 cells/5 fields) compared with other groups.

### Co-exposure to OVA + ZymA + H-ASD increased the levels of cytokines and chemokines in BALF

**Fig. 4:**
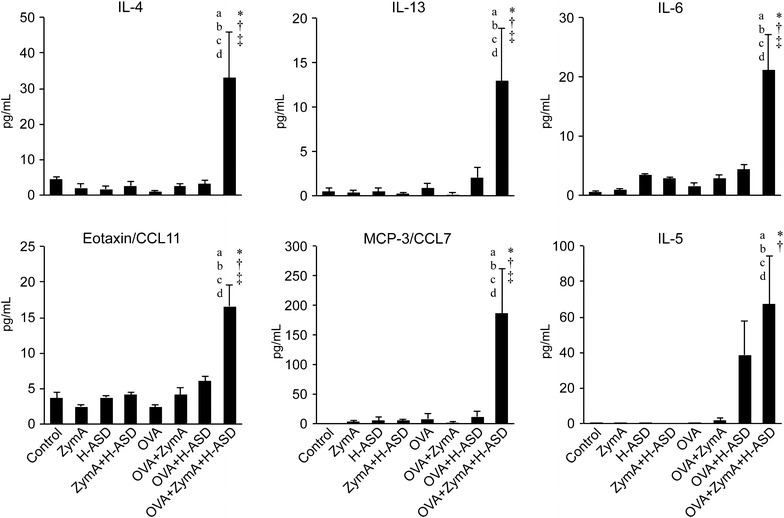
Effects of ZymA and H-ASD on the levels of IL-4, IL-13, IL-6, eotaxin/CCL11, MCP-3/CCL7, and IL-5 in BALF. All data are expressed as the mean ± SE, n = 8 per group.* a* p < 0.05 vs. control,* b* p < 0.05 vs. ZymA,* c* p < 0.05 vs. H-ASD,* d* p < 0.05 vs. ZymA + H-ASD, *p < 0.05 vs. OVA, ^†^p < 0.05 vs. OVA + ZymA, ^‡^p < 0.05 vs. OVA + H-ASD

To identify the mechanism underlying the immunological effects of ZymA and H-ASD on the allergic airway response during OVA-induced airway inflammation in mice, we analyzed the cytokine and chemokine levels in BALF. Protein levels of IL-4, IL-13, eotaxin/CCL11, and MCP-3/CCL7 in all experimental groups were almost the same as those in the control group, with the exception of the OVA + ZymA + H-ASD group (Fig. 4). Addition of H-ASD to OVA increased the level of IL-5 relative to OVA alone, but the change was not statistically significant. By contrast, the cytokine levels (IL-4, IL-13, IL-6, eotaxin/CCL11, MCP-3/CCL7, and IL-5) in the OVA + ZymA + H-ASD group were markedly increased relative to the OVA and OVA + ZymA groups. The levels of these cytokines were also elevated in comparison to the OVA + H-ASD group; in addition, we observed significant changes in IL-4, IL-13, IL-6, eotaxin/CCL11, and MCP-3/CCL7 levels.

**Fig. 5:**
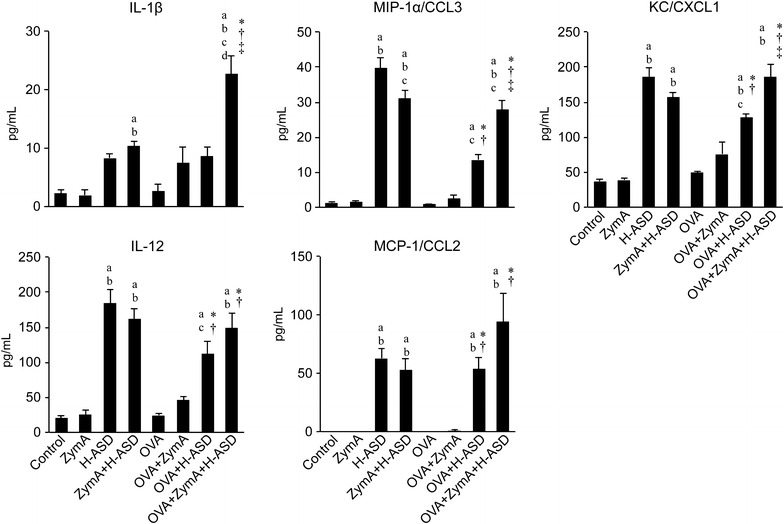
Effects of ZymA and H-ASD on the levels of IL-1β, MIP-1α/CCL3, KC/CXCL1, IL-12, and MCP-1/CCL2 in BALF. All data are expressed as the mean ± SE, n = 8 per group.* a* p < 0.05 vs. control,* b* p < 0.05 vs. ZymA,* c* p < 0.05 vs. H-ASD,* d* p < 0.05 vs. ZymA + H-ASD, *p < 0.05 vs. OVA, ^†^p < 0.05 vs. OVA + ZymA, ^‡^p < 0.05 vs. OVA + H-ASD

Moreover, the levels of IL-1β, MIP-1α/CCL3, and KC/CXCL1 were significantly greater in the OVA + ZymA + H-ASD than in the OVA, OVA + ZymA, and OVA + H-ASD groups (Fig. 5). The protein levels of IL-12 and MCP-1/CCL2 in BALF in the OVA + ZymA + H-ASD group were higher than those in the OVA + H-ASD group, albeit not at the level of statistical significance. In addition, the protein levels of MIP-1α/CCL3, KC/CXCL1, IL-12, and MCP-1/CCL2 in the H-ASD and ZymA + H-ASD groups were the same as or higher than those in the OVA + ZymA + H-ASD group. IFN-γ was not detected in this study.

### Co-exposure to ZymA and H-ASD increased the production of OVA-specific IgE in serum

**Fig. 6:**
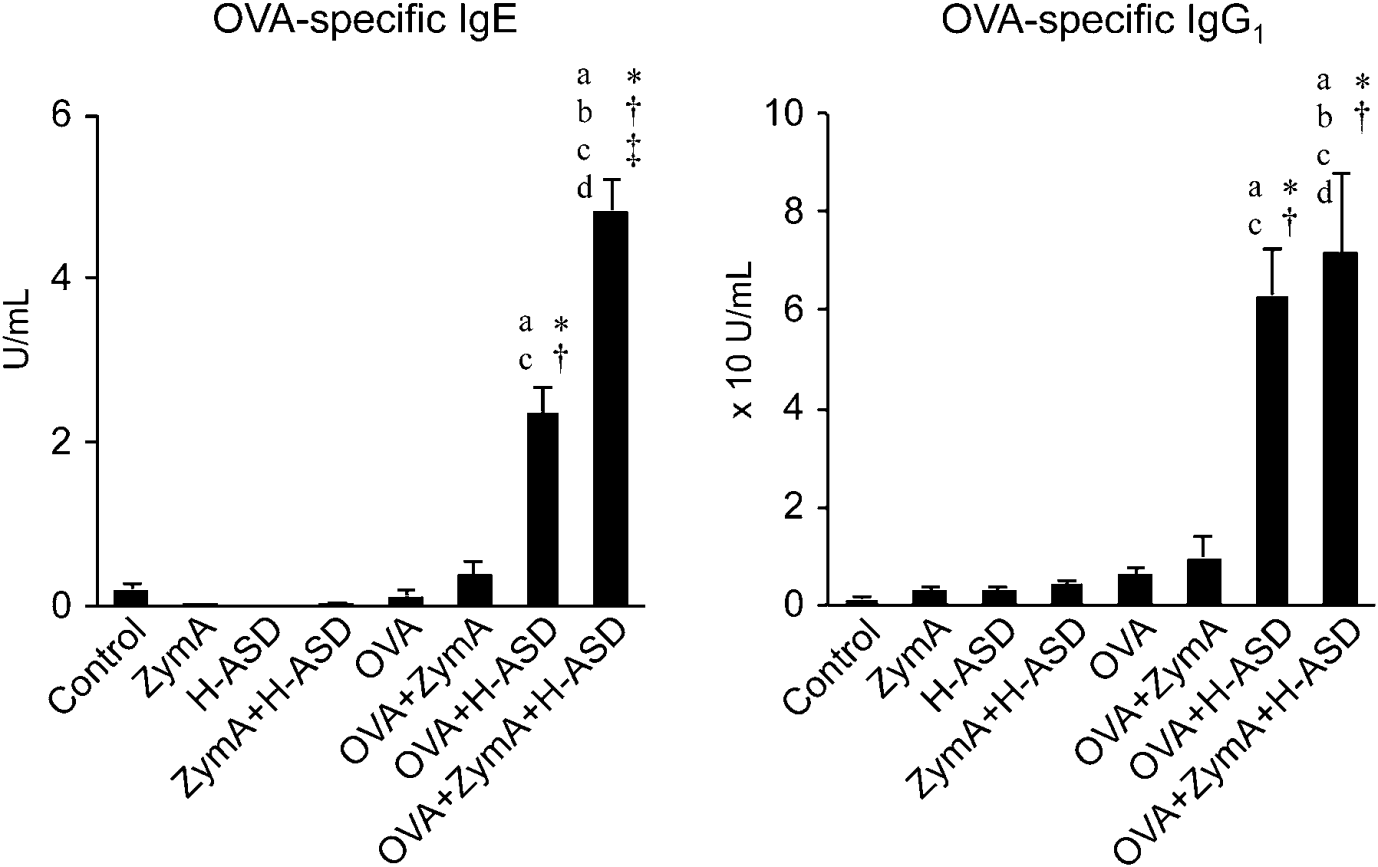
Effects of ZymA and H-ASD on the levels of OVA-specific antibodies in serum. According to the manufacturer’s protocol, 1 U/ml of anti-OVA IgE and anti-OVA IgG_1_ are defined as 1.3 ng/ml and 160 ng/ml of the antibody, respectively. All data are expressed as the mean ± SE, n = 14 per group.* a* p < 0.05 vs. control,* b* p < 0.05 vs. ZymA,* c* p < 0.05 vs. H-ASD,* d* p < 0.05 vs. ZymA + H-ASD, *p < 0.05 vs. OVA, ^†^p < 0.05 vs. OVA + ZymA, ^‡^p < 0.05 vs. OVA + H-ASD

The levels of IgE and IgG_1_ in the OVA and OVA + ZymA groups were almost the same as those in the control group (Fig. 6). Addition of H-ASD to OVA increased the production of these antibodies significantly. Moreover, the combination OVA + ZymA + H-ASD caused a marked upregulation of IgE but not of IgG_1_.

## Discussion

In this study, we demonstrated the exacerbating effects of the combined treatment of ZymA as a TLR2 ligand coupled with H-ASD on OVA-induced lung eosinophilia in a mouse model. ZymA alone had an effect similar to the control (saline), except for the macrophage number in BALF, which was significantly increased by ZymA. Treatment with ZymA + H-ASD caused a small increase in neutrophils in BALF in comparison with H-ASD alone. Cytokines and chemokines in BALF were not upregulated. The addition of ZymA to OVA did not increase the numbers of inflammatory cells and pro-inflammatory cytokines and chemokines over those of OVA alone. OVA + H-ASD significantly increased the protein levels of MIP-1α/CCL3, KC/CXCL1, IL-12, and MCP-1/CCL2 in BALF and OVA-specific antibodies in serum. Treatment with OVA + ZymA + H-ASD caused serious exacerbation of lung pathology: eosinophilic infiltration and production of excess mucus in the airway epithelium along with markedly greater numbers of inflammatory cells and Th2 cytokines (IL-4, IL-13, IL-5), other cytokines (IL-1β, IL-6), and chemokines (eotaxin/CCL11, MCP-3/CCL7) in BALF. These Th2 cytokines and chemokines are key mediators of the symptoms of asthma and are critical for the recruitment and survival of eosinophils [27]. Therefore, we conclude that the combination ZymA + H-ASD may be an important factor in exacerbating the effects of OVA-induced lung eosinophilia.

Some studies have shown fungi to be associated with the exacerbation of allergic respiratory diseases [28–30]. Therefore, it is likely that microorganisms such as fungi present in airborne ASD are associated with an increase in the incidence of allergic airway diseases from spring to early summer in East Asia. Our previous results have shown that the exacerbating effect of the fungus *B. adusta* is much stronger than the effects of other microorganisms, including gram-positive bacteria isolated from a wind-borne ASD aerosol collected on the Noto peninsula [31]. Although the composition of the fungal cell wall differs across various species, the cell wall skeleton in most fungi consists of cross-linked polysaccharides, β-1,3-glucan, chitin, and a surface glycoprotein [32, 33]. Some researchers have compared the pulmonary inflammatory potential of different components of the cell wall of yeasts and other fungi and concluded that β-1,3-glucan has more potent effects than chitin and mannan [34]. The current study suggests that β-1,3-glucan in ZymA associated with H-ASD may be the most likely culprit for exacerbation of OVA-induced murine lung eosinophilia.

In this study, we used the commercial polysaccharide ZymA, which is prepared from the cell wall of *S. cerevisiae*. β-Glucan present in ZymA is generally known to have a potent immunostimulatory activity and to act as an antiallergic agent. Oral administration of β-glucan prepared from *Ganoderma lucidum*, a medicinal mushroom, inhibited the production of Th2 cytokines (IL-4 and IL-5) by splenocytes and attenuated the upregulation of antigen-specific IgE in the serum of B6 mice sensitized with OVA intraperitoneally [35]. Intraperitoneal injection of curdlan, another linear β-1,3-glucan, also reduced the number of eosinophils and the level of Th2 cytokines (IL-5 and IL-13) in BALF in a mouse model of OVA-induced allergic airway inflammation [36]. As for humans, it has been reported that β-glucan has a possible antiallergic effect on patients with allergic diseases [37, 38]. In contrast, the exposure of the airway to β-glucan exacerbates the airway allergic responses. The level of Th2 cytokines (IL-4, IL-5, and IL-13) in BALF was increased by the intratracheal administration of soluble β-glucan from *Candida albican*s in a mouse model of allergic sensitization of the airway by OVA [39]. Sensitization of the airway with ZymA (1–75 µg) and OVA via pharyngeal aspiration increased the number of lung eosinophils and the levels of lung IL-5 and serum OVA-specific IgE in mice [40]. As mentioned above, β-glucan can exacerbate allergic airway disorders in murine models, and accordingly, we demonstrated in the present study that exposure of the mouse airway to much smaller doses of ZymA with H-ASD also exacerbated the OVA-induced allergic airway inflammation. In other studies, the dose was 1–75 µg of ZymA or 10–25 µg of β-glucan per animal, whereas in this study, 20 ng of ZymA per animal was used. The combination OVA + H-ASD caused slight exacerbation of the allergic airway inflammation. Therefore, this ZymA treatment dose can strongly elicit the potential of OVA + H-ASD to exacerbate allergic reactions in mice. We have reported similar evidence that trace LPS (1 ng) can elicit the potential of OVA + H-ASD to exacerbate lung eosinophilia in mice [24].

In addition, we used a small dose of OVA (4 µg/animal) in the present study. The combination OVA + ZymA + H-ASD induced the most severe exacerbation in murine lung eosinophilia despite the fact that the OVA alone group showed no significant difference as compared to the control group. Thus, this means that the treatment dose of OVA in this study can elucidate the potential of ZymA + H-ASD to exacerbate allergic reaction.

In the present study, we exposed model mice to four times 0.4 mg of H-ASD throughout the 2-week experimental period. This amount is equivalent to the amount of particles deposited into the lungs of mice exposed to air containing 7 mg/m^3^/day SPM for 6 weeks, which approximates the level of atmospheric contamination—consisting mainly of sand dust—in China during spring [41, 42].

TLRs were the first pattern recognition receptors reported that helped researchers recognize pathogen-derived molecular structures during innate immune responses invoked by microbial pathogens [43]. Recently, it was reported that the activation of innate immune responses via TLR–ligand binding plays an important role in the development of adaptive immune responses [44]. The activation of antigen-presenting cells, such as dendritic cells and macrophages, during adaptive immune responses requires pathogen recognition by TLR2 and TLR4 [45, 46]. We have reported that the production of pro-inflammatory cytokines caused by *B. adusta* was mediated by TLR2 rather than TLR4 in an in vitro experiment using bone marrow-derived macrophages from wild-type, TLR2 knockout, and TLR4 knockout mice [23]. Furthermore, we have shown that co-exposure to *B. adusta* and H-ASD exacerbated lung eosinophilia via the TLR2 signaling pathway [47]. Co-treatment with the TLR2 ligand Pam3Cys and OVA activated an OVA-associated Th2-based immune response in experimental asthma [48]. Hence, in the present study, we reasoned that the combination of ZymA and H-ASD might cause exacerbation of OVA-induced lung allergy via the TLR2-signaling pathway. Dectin-1 can recognize β-glucan in collaboration with TLR2. The binding of ligands to these receptors induces the production of proinflammatory cytokines, such as IL-1β, IL-6, and tumor necrosis factor-α via the nuclear factor κB pathway in macrophages [49, 50]. Although we did not investigate the association with dectin-1 and its role in the exacerbation of allergic airway inflammation through ASD exposure, dectin-1 may act in concert with TLR2 in this process. In addition, besides β-1,3-glucan, ZymA contains compounds such as mannan, chitin, and proteins [51]. Thus, although β-1,3-glucan is the main component of the fungal cell wall [34], other receptors (e.g., dectin-2 for mannan) might play a role in the exacerbation of lung eosinophilia.

Widely used particulate adjuvants such as alum (aluminum hydroxide) are considered to induce the activation of cellular innate immune responses and evoke the enhancement of acquired immune responses. Particles such as Alum and silica enhance the phagocytosis of innate cells and then induce Th2 activation through specific signaling pathways involving NACHT-, LRR-, and PYD domain-containing protein 3 (NLRP3) inflammasome and/or spleen tyrosine kinase [52–54]. ASD contains Al_2_O_3_ and crystalline silica. However, we have recently reported that the enhancement of the Th2-immune response by ASD is not through the NLRP3 inflammasome-pathway [47].

## Conclusions

This study showed that combined exposure to small doses of ZymA and H-ASD exacerbates allergen-induced lung eosinophilia. This finding supports the hypothesis that fungal elements such as β-glucan attached to ASD may contribute to the exacerbation of human asthma. Therefore, ASD-bound fungi, bacteria, and silica-carrying particulate matter may turn asymptomatic or mild asthma into more severe cases.

## Abbreviations

ASD: Asian sand dust
H-ASD: heat-inactivated ASD
OVA: ovalbumin
TLR: toll-like receptor
LPS: lipopolysaccharide
ZymA: zymosan A
BALF: bronchoalveolar lavage fluid
AB: alcian blue
ELISA: enzyme-linked immunosorbent assay
IL: interleukin
IFN: interferon
KC: keratinocyte chemoattractant
MCP: monocyte chemotactic protein
MIP: macrophage inflammatory protein
Ig: immunoglobulin
ANOVA: analysis of variance
Th2: T helper 2
NLRP3: NACHT-, LRR-, and PYD domain-containing protein 3
